# Association between thyroid-stimulating hormone (TSH) and proteinuria in relation to thyroid cyst in a euthyroid general population

**DOI:** 10.1186/s40101-021-00264-y

**Published:** 2021-10-09

**Authors:** Yuji Shimizu, Yuko Nabeshima-Kimura, Shin-Ya Kawashiri, Yuko Noguchi, Shigeki Minami, Yasuhiro Nagata, Takahiro Maeda, Naomi Hayashida

**Affiliations:** 1 Department of General Medicine, Nagasaki University Graduate School of Biomedical Sciences, Nagasaki, Japan; 2 Department of Cardiovascular Disease Prevention, Osaka Center for Cancer and Cardiovascular Diseases Prevention, Osaka, Japan; 3 Department of Community Medicine, Nagasaki University Graduate School of Biomedical Sciences, Nagasaki-shi, Sakamoto 1-12-4, Nagasaki, 852-8523 Japan; 4 Department of Radiation Health Management, Fukushima Medical University, Fukushima, Japan; 5 Department of Breast and Endocrine Surgery, Nagasaki Harbor Medical Center, Nagasaki, Japan; 6 Division of Promotion of Collaborative Research on Radiation and Environment Health Effects, Atomic Bomb Disease Institute, Nagasaki University, Nagasaki, Japan

**Keywords:** Euthyroid, Proteinuria, T3, T4, Thyroid cysts, TSH

## Abstract

**Background:**

High normal levels of thyroid-stimulating hormone (TSH) have been reported to be associated with chronic kidney disease (CKD) among euthyroid individuals. However, there has been only limited research on the association between TSH and proteinuria, a major risk factor for the progression of renal disease.

**Methods:**

A cross-sectional study of 1595 euthyroid individuals was conducted. All participants were within the normal range for free triiodothyronine (T3), free thyroxine (T4), and TSH. Analyses were stratified by thyroid cyst status to test the hypothesis that the absence of thyroid cysts, an indicator of latent thyroid damage, is associated with declining ability to synthesis thyroid hormone.

**Results:**

For participants with thyroid cysts, a significant inverse association between TSH and proteinuria was observed (adjusted odds ratio (95% confidence intervals) of log-transformed TSH for proteinuria 0.40 (0.18, 0.89)). In participants without thyroid cysts, a significant positive association between those two factors was observed (2.06 (1.09, 3.90)).

**Conclusions:**

Among euthyroid individuals in the general population, being in the normal range of TSH was found to have an ambivalent association with proteinuria. Thyroid cyst status could be an effect modifier for those associations.

## Introduction

High normal levels of thyroid-stimulating hormone (TSH) have been reported to be associated with decreased glomerular filtration rate (GFR) [1] and chronic kidney disease (CKD) in euthyroid individuals [2].

Proteinuria is a major risk factor for the progression of renal disease [3]. Therefore, unlike GFR, which indicates current renal function, proteinuria is a useful marker for evaluating the progression of renal injury. However, few studies have evaluated the association between TSH and proteinuria among euthyroid individuals. A hospital-based Saudi Arabian study with euthyroid patients with diabetes reported a positive association between TSH levels and the severity of albuminuria and an inverse association between free thyroxine (T4) levels and the severity of albuminuria [4]. Therefore, in euthyroid individuals, high normal TSH levels could be positively associated with proteinuria, partly by indicating low normal ability to synthesis thyroid hormone. In contrast, high ability to synthesis thyroid hormone might have a beneficial influence by preventing proteinuria.

Anti-thyroid peroxidase antibody (TPO-Ab) is inversely associated with thyroid cysts in individuals with normal thyroid function [5]. Since thyroid peroxidase plays an important role in thyroid hormone synthesis [6], latent damage to the thyroid caused by TPO-Abs might reduce thyroid cysts. Therefore, the absence of thyroid cysts might negatively affect the activation and production of thyroid hormone.

In addition, demands for thyroid hormone might decrease with aging [7]. Since individuals with thyroid cysts are older than individuals without thyroid cysts [8, 9], individual with thyroid cysts might have enough capacity to synthesize thyroid hormone based on demand as indicated by TSH levels. Therefore, individuals with thyroid cysts might have relatively higher thyroid hormone activity, as we have previously reported [8, 9].

Furthermore, thyroglobulin plays a crucial role in the activation and synthesis of free triiodothyronine (T3) and free T4 [10]. The fluid in thyroid cysts is rich in thyroglobulin [11]. Thus, the presence or absence of thyroid cysts could influence the association between TSH and proteinuria in the general population. Individuals with thyroid cysts might benefit from extra thyroglobulin. Thus, thyroid cysts might have a beneficial effect on thyroid hormone production.

Upon demand, TSH is secreted in the pituitary ground and activates the thyroid to increase peripheral free T3 and free T4 levels. Therefore, if the thyroid is functioning appropriately, TSH levels indicate the ability to synthesize thyroid hormone. However, if thyroid function declines, TSH levels indicate thyroid hormone deficiency, a risk factor for proteinuria.

Since individuals with thyroid cysts might benefit from the activation and production of thyroid hormones [8, 9], we hypothesized that being in the normal range of TSH is inversely associated with proteinuria among euthyroid individuals with thyroid cysts in the general population. We also hypothesized that being in the normal range of TSH is positively associated with proteinuria among euthyroid individuals without thyroid cysts in the general population.

The aim of the present study was to clarify the associations between TSH and proteinuria stratified by thyroid cyst status by studying a Japanese general population with free T3, free T4, and TSH levels in the normal range.

## Material and methods

### Study population

To clarify the influence of benign thyroid disease such as cysts and nodules on health, a thyroid survey was started in 2014. In 2014, 1883 individuals aged 40–74 years participated in this survey. Based on this survey, we found that thyroid cysts could have clinical relevance, even though they were generally regarded as clinically insignificant [5, 8, 9]. Since the present study is aimed to clarify one of the biological functions that thyroid cysts might possess, we used this thyroid survey, as in our previous studies [5, 8, 9].

In this cross-sectional study, the study population comprised 1883 Japanese individuals aged 40–74 years from the town of Saza in western Japan. These individuals underwent an annual medical check-up in 2014 as recommended by the Japanese government.

To avoid the influence of thyroid disease, individuals were excluded if they had a history of thyroid disease (*n* = 60); had missing data thyroid function such as TSH, free T3, and free T4 levels (*n* = 17); or who were out of the normal range for T3 (2.1–4.1 pg/mL), T4 (1.0–1.7 ng/dL) (*n* = 77). Individuals out of the normal range for TSH (0.39–4.01 μIU/mL) were also excluded (*n* = 126).

Individuals without data on body mass index (BMI) (*n* = 1) or blood pressure (*n* = 1) were also excluded. Individuals were also excluded if data from serum tests (*n* = 4) or drinking and smoking status (*n* = 2) were missing. The study included 1595 participants, with a mean age of 60.5 years (standard deviation (SD), 9.1; range 40–74).

### Data collection and laboratory measurements

Trained interviewers obtained information on clinical characteristics. Body weight and height were measured with an automatic body composition analyzer (BF-220; Tanita, Tokyo, Japan) to calculate BMI (kg/m^2^). Systolic blood pressure (SBP) and diastolic blood pressure (DBP) were recorded at rest.

A fasting blood sample was collected. TSH, free T3, and free T4 levels were measured using a chemiluminescent immunoassay (CLIA) at the LSI Medience Corporation (Tokyo, Japan). Normal ranges for free T3 (2.1–4.1 pg/mL), free T4 (1.0–1.7 ng/dL), and TSH (0.39–4.01 μIU/mL) based on this method were reported [12]. Hemoglobin A1c (HbA1c), triglyceride (TG), high-density lipoprotein cholesterol (HDLc), and serum creatinine levels were measured using standard procedures at SRL, Inc. (Tokyo, Japan).

The presence of thyroid cysts was determined by experienced ultrasound technicians using a LOGIQ Book XP with a 10-MHz transducer (GE Healthcare, Milwaukee, WI, USA). For the present study, a thyroid cyst was defined as a structure with a maximum diameter of ≥ 2.0 mm and no solid components [5, 8, 9].

Spot urine dipstick was used to assess for the presence of proteinuria. Proteinuria was diagnosed as +1 or above.

### Statistical analysis

In order to validate the study population in the present study, goodness of fit was evaluated using the Hosmer-Lemeshow test.

To evaluate the secretory capacity of the pituitary gland, Jostel’s TSH index (TSHI) was calculated [13]. To evaluate the thyroid’s secretory capacity and global step-up deiodinase activity, maximum thyroid secretory capacity (SPINA-GT) and the overall activity of peripheral deiodinases (SPINA-GD) were also calculated using the diagnostic application of SPINA Thyr 4.1 (SciCrunch, University of California, CA, USA) [14, 15].

Characteristics of the study population were expressed as means ± SD, except for sex, daily drinker or current smoker status, and TSH. Sex and daily drinker or current smoker status were expressed as percentages. Since TSH values had a skewed distribution, log-transformed TSH values were used for analysis. TSH values were expressed as medians [the first quartile, third quartile]. We also evaluated sex- and age-adjusted values of thyroid hormones (free T3 and free T4) associated with the status of thyroid cysts using analysis of covariance (ANCOVA).

Logistic regression was used to calculate odds ratios (ORs) and 95% confidence intervals (CIs) to determine the association between proteinuria and thyroid cysts and between proteinuria and TSH.

Two adjustment models were used. Model 1 adjusted for sex and age. Model 2 adjusted for variables in model 1 and potential confounding factors directly associated with thyroid function: BMI (kg/m^2^), smoking status (never, former, current), drinking status (none, often, daily), SBP (mmHg), HbA1c (%), TG (mg/dL), and HDLc (mg/dL). We also stratified analyses by thyroid cyst status. For sensitivity analysis, we conducted sex-specific analyses using age-adjusted models. We also evaluated associations between log-transformed TSH values and proteinuria in participants without reduced renal function (defined as GFR ≥ 60 mL/min/1.73 m^2^). All statistical analyses were performed with SAS for Windows (version 9.4: SAS Inc., Cary, NC, USA). *P* values < 0.05 were regarded as statistically significant.

## Results

Among 1595 study participants, 525 (32.9%) were diagnosed as having thyroid cysts and 86 (5.4%) were diagnosed with proteinuria.

### Clinical characteristics of the study participants by thyroid cyst status

Table 1 shows the clinical characteristics of the study participants by thyroid cyst status. Compared to participants without thyroid cysts, participants with thyroid cysts were significantly less likely to be male and current smokers. They were significantly older and had significantly higher SBP and DBP.

**Table 1:** Characteristics of study population

	Thyroid cyst		*p*
	(−)	(+)	
No. of participants	1070	525	
Men, %	40.6	28.8	< 0.001
Postmenopausal women, %	44.7	59.8	< 0.001
Age, year	59.7 ± 9.4	62.0 ± 8.2	< 0.001
free T3, pg/mL	3.18 ± 0.32	3.15 ± 0.31	0.079
free T4, ng/dL	1.26 ± 0.16	1.24 ± 0.15	0.117
TSH, μIU/mL	1.52 [1.09, 2.14]^*1^	1.54 [1.07, 2.16]^*1^	0.689^*2^
TSHI	2.59 ± 0.52	2.57 ± 0.53	0.648
SPINA-GT, pmol/s	3.7 ± 1.4	3.7 ± 1.3	0.497
SPINA-GD, nmol/s	28.3 ± 3.8	28.3 ± 3.9	0.963
BMI, kg/m^2^	22.8 ± 3.3	22.6 ± 3.4	0.414
SBP, mmHg	124 ± 17	126 ± 17	0.003
DBP, mmHg	73 ± 11	74 ± 10	0.032
Current smoker, %	15.4	11.6	0.041
Daily drinker, %	41.2	39.6	0.542
HbA1c, %	5.6 ± 0.6	5.6 ± 0.5	0.055
TG, mg/dL	107 ± 81	101 ± 60	0.157
HDLc, mg/dL	60 ± 15	62 ± 15	0.110
GFR, mL/min/1.73 m^2^	71.8 ± 13.2	71.5 ± 12.7	0.633

*T3* triiodothyronine, *T4* thyroxine, *TSH* thyroid-stimulating hormone, *TSHI* Jostel’s TSH index, *SPINA-GT* maximum thyroid secretory capacity, *SPINA-GD* overall activity of peripheral deiodinases, *BMI* body mass index, *SBP* systolic blood pressure, *DBP* diastolic blood pressure, *HbA1c* hemoglobin A1c, *TG* triglycerides, *HDLc* high-density lipoprotein cholesterol, *GFR* glomerular filtration rate. Values are mean ± standard deviation

*1: Values are median [the first quartile, third quartile]. Regression model for mean values was used for determining *p* values.

*2: Logarithmic transformation was used for evaluating *p*

To avoid the influence of sex and age, adjusted values for free T3 and free T4 values in participants with and without thyroid cysts were calculated. The least square mean (standard error) free T3 was 3.17 (0.01) pg/mL in participants with thyroid cysts and 3.17 (0.001) pg/mL in participants without thyroid cysts (*p* = 0.980), whereas the least square mean (standard error) free T4 was 1.25 (0.004) ng/dL and 1.25 (0.01) ng/dL (*p* = 0.530), respectively.

### Associations between proteinuria and thyroid cyst status

Table 2 shows ORs and 95% CIs for proteinuria by thyroid cyst status. No significant associations were observed. Goodness of fit was validated for the present study population.

**Table 2:** Odds ratios (ORs) and 95% confidence intervals (CIs) of proteinuria in relation to thyroid cyst

	Thyroid cyst		*p*	*p* (goodness of fit test)
	(−)	(+)		
No. of participants	1070	525		
No. of proteinuria cases (%)	56 (5.2)	30 (5.7)		
Model 1	Ref	1.24 (0.78, 1.98)	0.367	0.475
Model 2	Ref	1.27 (0.76, 1.97)	0.400	0.975

*Ref* reference. Model 1: adjusted for sex and age. Model 2: + BMI, smoking status (never, former, current), drinking status (none, often, daily), systolic blood pressure (SBP), triglycerides (TG), high-density lipoprotein cholesterol (HDLc), and hemoglobin A1c (HbA1c)

### Association between proteinuria and TSH

Table 3 shows ORs and 95% CIs for proteinuria by TSH value. Overall, a slight, but not statistically significant, positive association between TSH level and proteinuria was observed. When we stratified by thyroid cyst status, different associations were observed. A significant positive association was observed for participants without thyroid cysts, whereas a significant inverse association was observed for participants with thyroid cysts. Goodness of fit was validated for the present study population stratified by thyroid cyst status.

**Table 3:** Odds ratios (ORs) and 95% confidence intervals (CIs) of proteinuria in relation to thyroid-stimulating hormone (TSH) by thyroid cyst

	TSH levels			*p*	Log TSH	*p* (goodness of fit test)
	(Low)	(Medium)	(High)			
**Total**						
No. of participants	526	539	530			
No. of proteinuria cases (%)	25 (4.8)	30 (5.6)	31 (5.8)			
Model 1	Ref	1.21 (0.70, 2.08)	1.32 (0.76, 2.27)	0.325	1.13 (0.70, 1.81)	0.933
Model 2	Ref	1.19 (0.68, 2.07)	1.35 (0.77, 2.36)	0.296	1.14 (0.70, 1.86)	0.370
**Thyroid cyst (−)**						
No. of participants	344	374	352			
No. of proteinuria cases (%)	9 (2.6)	22 (5.9)	25 (7.1)			
Model 1	Ref	2.39 (1.08, 5.26)	3.06 (1.40, 6.69)	0.005	2.10 (1.13, 3.90)	0.135
Model 2	Ref	2.38 (1.07, 5.30)	3.08 (1.38, 6.86)	0.006	2.06 (1.09, 3.90)	0.647
**Thyroid cyst (+)**						
No. of participants	182	165	178			
No. of proteinuria cases (%)	16 (8.8)	8 (4.8)	6 (3.4)			
Model 1	Ref	0.55 (0.23, 1.34)	0.37 (0.14, 0.98)	0.037	0.41 (0.18, 0.89)	0.842
Model 2	Ref	0.54 (0.21, 1.38)	0.34 (0.12, 0.94)	0.031	0.40 (0.18, 0.89)	0.849

*Ref* reference. Model 1: adjusted for sex and age. Model 2: + BMI, smoking status (never, former, current), drinking status (none, often, daily), systolic blood pressure (SBP), triglycerides (TG), high-density lipoprotein cholesterol (HDLc), and hemoglobin A1c (HbA1c). Tertile values of TSH for men and women are < 1.19 μIU/mL and < 1.24 μIU/mL for (low), 1.19‑1.82 μIU/mL and 1.24‑1.96 μIU/mL for (medium), and 1.83 μIU/mL ≤ and 1.97 μIU/mL ≤ for (high)

Investigations of associations between log-transformed TSH, thyroid cyst status, and proteinuria revealed a significant interaction. The adjusted *p* values for this interaction were 0.001 in both the sex- and age-adjusted model and the fully adjusted model.

For sensitivity analysis, we conducted sex-specific analysis by using an age-adjusted model. We found essentially the same associations. The adjusted ORs and 95% CIs for proteinuria by log-transformed TSH in participants with and without thyroid cysts, respectively, were 0.73 (0.18, 2.98) and 1.84 (0.74, 4.58) for men. The corresponding values for women were 0.31 (0.12, 0.83) and 2.36 (1.02, 5.48).

### Association between proteinuria and TSH among participants without reduced renal function

Results of additional analysis limited to participants without reduced renal function are shown in Table 4. We found essentially the same associations as among all participants. A significant positive association between proteinuria and log-transformed TSH was observed for participants without thyroid cysts, whereas a significant inverse association was observed for participants with thyroid cysts.

**Table 4:** Odds ratios (ORs) and 95% confidence intervals (CIs) of proteinuria in relation to thyroid-stimulating hormone (TSH) by thyroid cyst among subjects without reduced renal function

	Total		Thyroid cyst (−)		Thyroid cyst (+)	
	Log TSH	*p*	Log TSH	*p*	Log TSH	*p*
No. of participants	1327		898		429	
No. of proteinuria cases (%)	66 (5.0)		41 (4.6)		25 (5.8)	
Model 1	1.12 (0.65, 1.92)	0.688	2.38 (1.15, 4.91)	0.020	0.35 (0.14, 0.86)	0.022
Model 2	1.13(0.64, 1.98)	0.678	2.35 (1.11, 5.00)	0.026	0.33 (0.13, 0.85)	0.021

*Ref* reference. Model 1: adjusted for sex and age. Model 2: + body mass index (BMI), smoking status (never, former, current), drinking status (none, often, daily), systolic blood pressure (SBP), triglycerides (TG), high-density lipoprotein (HDLc), and hemoglobin A1c (HbA1c)

Investigations of the associations between log-transformed TSH and thyroid cyst status and proteinuria revealed a significant interaction. The adjusted *p* values for this interaction were 0.001 in the sex- and age-adjusted model and < 0.001 in the fully adjusted model. Goodness of fit was validated for the present study population stratified by thyroid cyst status (*p* ≥ 0.05) (not shown in table).

## Discussion

The major finding of the present study with euthyroid participants from a general population was that there is a significant inverse association between TSH and proteinuria in individuals with thyroid cysts and a significant positive association between those two factors in individuals without thyroid cysts. Thus, thyroid cyst status could affect the association between TSH and proteinuria.

Among euthyroid individuals, high normal levels of TSH have been reported to be associated with decreased renal function [1, 2]. A previous case-control study with 159 participants with renal disease and proteinuria and 900 control participants showed that participants with proteinuria have higher TSH values than controls [16]. On the other hand, a large cross-sectional study with 74,356 participants aged ≥ 20 years found that subclinical hypothyroidism is a novel risk factor for reduced renal function but not proteinuria [17]. These findings are compatible with our present results, which show a small positive association between TSH and proteinuria among participants overall, but it was not statistically significant.

Thyroid hormones play a protective role in the kidney through the inhibition of nuclear factor-κB pathways, possibly by affecting the pro-inflammation and anti-inflammation balance that affects the development of CKD [18]. Another study reported that thyroid hormone plays a direct role in endothelial repair [19, 20]. Since endothelial dysfunction is a well-known cause of CKD [21], higher thyroid hormone activity could have a beneficial effect in preventing CKD.

Proteinuria is a major risk factor of renal disease progression [3]. Proteinuria of increasing severity is associated with a faster rate of renal decline [22]. Thus, the severity of proteinuria could be useful for evaluating the protective effect of thyroid hormones against kidney injury.

TSH, by definition, stimulates the secretion of thyroid hormones. In the present study, for participants with thyroid cysts, TSH was found to be significantly inversely associated with proteinuria. For participants without thyroid cysts, TSH was found to be significantly positively associated with proteinuria. Because similar TSHI values were observed between euthyroid participants with and without thyroid cysts, pituitary function is not the best explanation for these paradoxical results.

Previously, TPO-Ab titers were revealed to be inversely associated with thyroid cysts among 1432 Japanese individuals with normal thyroid function (defined by being in the normal range for free T3 and free T4). When adjusted for known cardiovascular risk factors, the OR and 95% CI for thyroid cyst with respect to log-transformed TPO-Ab titer was 0.67 (0.58, 0.78) [5]. Since TPO-Ab causes autoimmune thyroid disease [23], latent damage of the thyroid might be associated with the absence of thyroid cysts. In addition, being in the normal range for TPO-Ab titer was positively associated with atherosclerosis among 1087 euthyroid Japanese individuals; the adjusted OR and 95% CI for atherosclerosis with respect to log-transformed TPO-Ab titer was 2.65 (1.27, 5.51) [24]. Since endothelial dysfunction is recognized as a mechanism that leads to glomerular injury and atherosclerosis [25], atherosclerosis is positively associated with CKD [26]. Therefore, latent damage of the thyroid might influence the progression of renal disease.

However, in the present study, similar SPINA-GT and SPINA-GD were observed in individuals with versus without thyroid cysts. All participants in this study were within the normal range for free T3, free T4, and TSH. Therefore, different levels of thyroid hormone demand between euthyroid individuals with and without thyroid cysts might be the basis for those associations.

Energy that is necessary for living is mainly produced in the mitochondria. Growth differentiation factor-15 (GDF-15), a potential biomarker of mitochondrial dysfunction, is elevated in aging and age-related disorders [27, 28]. However, even when thyroid hormone activates energy production, serum GDF-15 levels were elevated in patients with hyperthyroidism [29]. Therefore, age-related declines in energy production might stimulate production of thyroid hormone.

On the other hand, since decreased thyroid function may lead to extended longevity [7], aging processes decrease the demand on thyroid hormone activity. Age-related declines in physical activity reduce the demands for thyroid hormone. In addition, age-related declines in the demand for thyroid hormone might reduce the production of thyroid hormone.

Therefore, to maintain energy homeostasis and to adapt to age-related physical changes, the thyroid might make fine adjustments within the normal range of thyroid hormone and TSH levels. Thus, thyroid hormone demand, rather than serum levels of thyroid hormone and TSH, could be an important determinant for this adjustment.

Individuals with thyroid cysts are typically older than those without thyroid cysts [8, 9, 30, 31]. Therefore, thyroid cysts could be formed during the aging process, which is related to decreasing demand for thyroid hormone [30, 31]. Since thyroid cyst fluid is rich in thyroglobulin [11], thyroid cysts might act as storage of thyroglobulin. Thus, thyroid cysts might have a beneficial influence on thyroid hormone secretion when necessary [8, 9, 30, 31].

Furthermore, in our previous study of 1020 participants in the normal range for free T3 and free T4 and not taking anti-hypertensive medications, the presence of a thyroid cyst was positively associated with isolated systolic hypertension but not with isolated diastolic hypertension; the fully adjusted OR and 95% CI was 1.78 (1.12, 2.85) for isolated systolic hypertension and 0.81 (0.21, 3.16) for isolated diastolic hypertension [9]. Since hyperthyroidism is a major secondary cause of isolated systolic hypertension [32], euthyroid participants with thyroid cysts might have relatively higher thyroid hormone activity than those without thyroid cysts. This association also might indicate that age-related declines in the demand for thyroid hormone, which is related to the presence of thyroid cysts, could have a beneficial influence on thyroid hormone activity.

In addition, our previous study with 1724 euthyroid participants (i.e., individuals within the normal range of free T3 and free T4) also revealed that for those without thyroid cysts, TSH is significantly positively associated with hypertension [fully adjusted OR, 1.27; 95% CI (1.01, 1.61)] but this association was not observed for individuals with thyroid cysts [0.79 (0.57, 1.09)] [8]. Since euthyroid participants with thyroid cysts might have comparatively higher ability to synthesize thyroid hormone than those without thyroid cysts [8, 9] and hypothyroidism is a recognized cause of secondary hypertension [33], the study findings partly support our hypothesis that TSH levels might indicate thyroid hormone deficiency among individuals without thyroid cysts.

Since thyroid cysts might have a beneficial influence on activating thyroid hormone synthesis [8, 9] among individuals with thyroid cysts, TSH levels might indicate thyroid hormone activity that has a protective effect against renal injury. Among individuals without thyroid cysts, TSH levels indicate thyroid hormone deficiency. Among individuals with thyroid cysts, an inverse association between TSH and proteinuria was observed. In contrast, among individuals without thyroid cysts, a positive association between TSH and proteinuria was observed.

Since higher thyroid hormone activity could prevent the progression of CKD [18‑21], thyroid cysts could be inversely associated with proteinuria and reflect the absence of latent thyroid damage. However, in the present study, no significant associations between thyroid cyst status and proteinuria were observed.

Aging is a well-known risk factor for cardiovascular disease and reduced renal function. Polycystic thyroid disease (PCTD) is a rare condition that is reported to be associated with subclinical or overt hypothyroidism. PCTD is more common in older individuals [34]. Since aging also might reduce the necessity of thyroid hormone activity [7] and might be associated with thyroid cyst development [8, 9, 30, 31], biological aging might be an important consideration while evaluating the influence of thyroid hormone and thyroid cysts on proteinuria.

Levels of GDF-15, which is considered a marker of biological age [27], were elevated in individuals with hyperthyroidism [29]. Since higher GDF-15 levels are associated with renal outcomes [35], biological age could be an important determinant of the association between TSH and proteinuria. Further investigation with data on GDF-15 is necessary to clarify those associations.

From a clinical perspective, the present study indicates that thyroid cysts could play an important beneficial role in thyroid hormone synthesis among participants with reduced demand of thyroid hormone. We evaluated thyroid function in a euthyroid population, i.e., individuals within the normal ranges for TSH, free T3, and free T4. We concluded that those three factors are not sufficient for evaluating thyroid function. Furthermore, in evaluating mortality, proteinuria has been reported to be more useful than GFR, especially in participants with higher GFR levels [36–38]. When we limited our analysis to subjects without reduced renal function, we found that TSH has a positive association with proteinuria in participants without thyroid cysts and an inverse association in participants with thyroid cysts. These results could be useful in clarifying the mechanism underlying age-related changes in thyroid function and provide a novel strategy for reducing the risk of mortality associated with proteinuria.

This study has potential limitations that warrant consideration. First, we thought that thyroid cysts might influence the activity of thyroid hormones but not their plasma concentrations. However, we were unable to evaluate thyroid hormone activity with precision in this study. Since nitric oxide could act as an indicator of thyroid replacement therapy [39], further investigation with a marker of oxidative stress such as nitric oxide is necessary. Second, although fluid from thyroid cysts is rich in thyroglobulin, the presence of anti-thyroglobulin antibodies decreases thyroglobulin levels dramatically [11]. Thus, anti-thyroglobulin antibodies might act as a strong confounding factor in our analysis. However, because of the limited volume of blood samples, we could not evaluate the influence of anti-thyroglobulin antibodies. The presence of anti-thyroglobulin antibodies might have a strong confounding effect, weakening the association between TSH and proteinuria. However, in the present study, significant associations in opposing directions were found between TSH and proteinuria according to thyroid cyst status. We evaluated the presence or absence of thyroid cysts. However, the number of cysts and the size of a given cyst could be important factors that affect the influence of thyroid cysts on thyroid hormone activity since PCTD is reported to be associated with hypothyroidism [34]. Further investigation is necessary. In this study, proteinuria was diagnosed using a urine dipstick test. To evaluate the precise risk for renal disease progression, quantitative tests for markers such as urinary albumin level are necessary. Finally, because this was a cross-sectional study, no causal relationship could be established. Further investigation with a longitudinal study is necessary.

## Conclusions

In conclusion, among euthyroid individuals, being in the normal range of TSH is inversely associated with proteinuria among individuals with thyroid cysts, but positively associated with proteinuria in individuals without thyroid cysts. The presence of thyroid cysts could be an effect modifier for the association between TSH and proteinuria.

## Data Availability

We cannot publicly provide individual data due to participant privacy, according to ethical guidelines in Japan. Additionally, the informed consent obtained does not include a provision for publicity sharing data. Qualifying researchers may apply to access a minimal dataset by contacting Prof Naomi Hayashida, Principal Investigator, Division of Promotion of Collaborative Research on Radiation and Environment Health Effects, Atomic Bomb Disease Institute, Nagasaki University, Nagasaki, Japan, at naomin@nagasaki-u.ac.jp. Or, please contact the office of data management at ritouken@vc.fctv-net.jp. Information for where data request is also available at https://www.genken.nagasaki-u.ac.jp/dscr/message/ and http://www.med.nagasaki-u.ac.jp/cm/.

## Abbreviations

TSH: Thyroid-stimulating hormone
CKD: Chronic kidney disease
T3: Triiodothyronine
T4: Thyroxine
GFR: Glomerular filtration rate
TPO-Ab: Anti-thyroid peroxidase antibody
BMI: Body mass index
SD: Standard deviation
SBP: Systolic blood pressure
DBP: Diastolic blood pressure
CLIA: Chemiluminescent immunoassay
HbA1c: Hemoglobin A1c
TG: Triglyceride
HDLc: High-density lipoprotein cholesterol
TSHI: Jostel’s TSH index
SPINA-GT: Maximum thyroid secretory capacity
SPINA-GD: Overall activity of peripheral deiodinases
GDF-15: Growth differentiation factor-15
ANCOVA: Analysis of covariance
ORs: Odds ratios
CIs: Confidence intervals
PCTD: Polycystic thyroid disease

## References

[1] Tanaka Y, Furusyo N, Kato Y, Ueyama T, Yamasaki S, Ikezaki H, Murata M, Hayashi J (2018). Correlation between thyroid stimulating hormone and renal function in euthyroid residents of Japan: results from the Kyushu and Okinawa Population Study (KOPS). J Atheroscler Thromb..

[2] Zhang Y, Chang Y, Ryu S, Cho J, Lee WY, Rhee EJ, Kwon MJ, Pastor-Barriuso R, Rampal S, Han WK, Shin H, Guallar E (2014). Thyroid hormone levels and incident chronic kidney disease in euthyroid individuals: the Kangbuk Samsung Health Study. Int J Epidemiol..

[3] Cravedi P, Remuzzi G (2013). Pathophysiology of proteinuria and its value as an outcome measure in chronic kidney disease. Br J Clin Pharmacol..

[4] Aljabri KS, Alnasser IM, Facharatz, Bokhari SA, Alshareef MA, Khan PM, Mallosho AM, AbuElsaoud HM, Jalal MM, Safwat RF, Boraie RE, Aljabri NK, Aljabri BK, Alsuraihi AY, Hawsawi AI (2019). Association of serum thyroid stimulating hormone and free thyroxine with urinary albumin excretion in euthyroid subjects with type 2 diabetes mellitus. Int J Diabetes Clin Res.

[5] Shimizu Y, Nabeshima-Kimura Y, Kawashiri SY, Noguchi Y, Nagata Y, Maeda T, Hayashida N (2020). Anti-thyroid peroxidase antibody and thyroid cysts among the general Japanese population: a cross-sectional study. Environ Health Prev Med..

[6] Godlewska M, Banga PJ (2019). Thyroid peroxidase as a dual active site enzyme: focus on biosynthesis, hormonogenesis and thyroid disorders of autoimmunity and cancer. Biochimie..

[7] Gesing A, Lewiński A, Karbownik-Lewińska M (2012). The thyroid gland and the process of aging; what is new?. Thyroid Res..

[8] Shimizu Y, Nabeshima-Kimura Y, Kawashiri SY, Noguchi Y, Nagata Y, Maeda T, Hayashida N (2020). Associations between thyroid-stimulating hormone and hypertension according to thyroid cyst status in the general population: a cross-sectional study. Environ Health Prev Med..

[9] Shimizu Y, Kawashiri SY, Noguchi Y, Nagata Y, Maeda T, Hayashida N (2020). Anti-thyroid peroxidase antibody and subclinical hypothyroidism in relation to hypertension and thyroid cysts. PLoS One..

[10] Citterio CE, Targovnik HM, Arvan P (2019). The role of thyroglobulin in thyroid hormonogenesis. Nat Rev Endocrinol..

[11] Salabè GB, Fusco A, Milani C, Baschieri I, Ventura T, Cortiello M (1990). Identification of serum proteins, thyroglobulin and antithyroid antibodies in the fluid of thyroid cysts. Thyroidology..

[12] LSI Medience Corporation Information. Rinsyokensa Jugyo. 2017;17-04. [Cited 5^th^ Oct 2021]. Available from: https://www.medience.co.jp/clinical/information/parts/pdf/17-04.pdf.

[13] Jostel A, Ryder WD, Shalet SM (2009). The use of thyroid function tests in the diagnosis of hypopituitarism: definition and evaluation of the TSH index. Clin Endocrinol (Oxf).

[14] Dietrich JW, Landgrafe G, Fotiadou EH (2012). TSH and thyrotropic agonists: key actors in thyroid homeostasis. J Thyroid Res.

[15] Dietrich JW, Landgrafe-Mende G, Wiora E, Chatzitomaris A, Klein HH, Midgley JE, Hoermann R (2016). Calculated parameters of thyroid homeostasis: emerging tools for differential diagnosis and clinical research. Front Endocrinol (Lausanne).

[16] Gilles R, den Heijer M, Ross AH, Sweep FC, Hermus AR, Wetzels JF (2008). Thyroid function in patients with proteinuria. Neth J Med..

[17] Chang YC, Chang CH, Yeh YC, Chuang LM, Tu YK (2018). Subclinical and overt hypothyroidism is associated with reduced glomerular filtration rate and proteinuria: a large cross-sectional population study. Sci Rep..

[18] Furuya F, Ishii T, Tamura S, Takahashi K, Kobayashi H, Ichijo M, Takizawa S, Kaneshige M, Suzuki-Inoue K, Kitamura K (2017). The ligand-bound thyroid hormone receptor in macrophages ameliorates kidney injury via inhibition of nuclear factor-κB activities. Sci Rep..

[19] Zhang L, Cooper-Kuhn CM, Nannmark U, Blomgren K, Kuhn HG (2010). Stimulatory effects of thyroid hormone on brain angiogenesis in vivo and in vitro. J Cereb Blood Flow Metab..

[20] Shakoor SK, Aldibbiat A, Ingoe LE, Campbell SC, Sibal L, Shaw J, Home PD, Razvi S, Weaver JU (2010). Endothelial progenitor cells in subclinical hypothyroidism: the effect of thyroid hormone replacement therapy. J Cin Endocrinol Metab..

[21] Goligorsky MS (2015). Pathogenesis of endothelial cell dysfunction in chronic kidney disease: a retrospective and what the future may hold. Kidney Res Clin Pract..

[22] Turin TC, James M, Ravani P, Tonelli M, Manns BJ, Quinn R, Jun M, Klarenbach S, Hemmelgarn BR (2013). Proteinuria and rate of change in kidney function in a community-based population. J Am Soc Nephrol..

[23] Carlé A, Laurberg P, Knudsen N, Perrild H, Ovesen L, Rasmussen LB, Jorgensen T, Pedersen IB (2006). Thyroid peroxidase and thyroglobulin auto-antibodies in patients with newly diagnosed overt hypothyroidism. Autoimmunity..

[24] Shimizu Y, Kawashiri SY, Noguchi Y, Nagata Y, Maeda T, Hayashida N (2020). Normal range of anti-thyroid peroxidase antibody (TPO-Ab) and atherosclerosis among eu-thyroid population: a cross-sectional study. Medicine (Baltimore).

[25] Endemann DH, Schiffrin EL (2004). Endothelial dysfunction. J Am Soc Nephrol..

[26] Shimizu Y, Yamanashi H, Noguchi Y, Koyamatsu J, Nagayoshi M, Kiyoura K, Fukui S, Tamai M, Kawashiri SY, Kondo H, Maeda T (2019). Association between chronic kidney disease and carotid intima-media thickness in relation to circulating CD34-positive cell count among community-dwelling elderly Japanese men. Atherosclerosis..

[27] Conte M, Ostan R, Fabbri C, Santoro A, Guidarelli G, Vitale G, Mari D, Sevini F, Capri M, Sandri M, Monti D, Franceschi C, Salvioli S (2019). Human aging and longevity are characterized by high levels of mitokines. J Gerontol A Biol Sci Med Sci..

[28] Fujita Y, Taniguchi Y, Shinkai S, Tanaka M, Ito M. Secreted growth differentiation factor 15 as a potential biomarker for mitochondrial dysfunctions in aging and age-related disorders. Geriatr Gerontol Int. 2016;(Suppl 1):17–29. 10.1111/ggi.12724 27018280

[29] Zhao J, Li M, Chen Y, Zhang S, Ying H, Song Z, Lu Y, Li X, Xiong X, Jiang J (2019). Elevated serum growth differentiation factor 15 levels in hyperthyroid patients. Front Endocrinol (Lausanne).

[30] Shimizu Y, Kawashiri SY, Noguchi Y, Nagata Y, Maeda T, Hayashida N (2021). Association between thyroid cysts and hypertension by atherosclerosis status: a cross-sectional study. Sci Rep..

[31] Shimizu Y, Kawashiri SY, Noguchi Y, Nagata Y, Maeda T, Hayashida N (2021). HbA1c is inversely associated with thyroid cysts in a euthyroid population: a cross-sectional study. PLoS One..

[32] Prisant LM, Gujral JS, Mulloy AL (2006). Hyperthyroidism: a secondary cause of isolated systolic hypertension. J Clin Hypertens (Greenwich).

[33] Stabouli S, Papakatsika S, Kotsis V (2010). Hypothyroidism and hypertension. Expert Rev Cardiovasc Ther..

[34] Kubota S, Maruta T, Fujiwara M, Hagiwara H, Tsujimoto N, Kudo T, Nishihara E, Ito M, Amino N, Miyauchi A (2010). The prevalence of polycystic thyroid disease in hypothyroid patients with negative thyroid autoantibodies. Thyroid..

[35] Ho JE, Hwang SJ, Wollert KC, Larson MG, Cheng S, Kempf T, Vasan RS, Januzzi JL, Wang TJ, Fox CS (2013). Biomarkers of cardiovascular stress and incident chronic kidney disease. Clin Chem..

[36] Matsushita K, van der Velde M, Astor BC, Woodward M, Levey AS, de Jong PE, Coresh J, Gansevoort RT, Chronic Kidney Disease Prognosis Consortium (2010). Association of estimated glomerular filtration rate and albuminuria with all-cause and cardiovascular mortality in general population cohorts: a collaborative meta-analysis. Lancet.

[37] Gansevoort RT, de Jong PE (2010). Challenges for the present CKD classification system. Curr Opin Nephrol Hypertens..

[38] Gansevoort RT, Matsushita K, van der Velde M, Astor BC, Woodward M, Levey AS, de Jong PE, Coresh J, Chronic Kidney Disease Prognosis Consortium (2011). Lower estimated GFR and higher albuminuria are associated with adverse kidney outcomes. A collaborative meta-analysis of general and high-risk population cohorts. Kidney Int..

[39] Obradovic M, Gluvic Z, Sudar-Milovanovic E, Panic A, Trebaljevac J, Bajic V, Zarkovic M, Isenovic ER (2016). Nitric oxide as a marker for levo-thyroxine therapy in subclinical hypothyroid patients. Curr Vasc Pharmacol..

